# The ETS Family Member TEL Binds to Nuclear Receptors RAR and RXR and Represses Gene Activation

**DOI:** 10.1371/journal.pone.0023620

**Published:** 2011-09-16

**Authors:** Magda A. Meester-Smoor, Marjolein J. F. W. Janssen, W. Martijn ter Haar, Karel H. M. van Wely, Albert-Jan L. H. J. Aarnoudse, Gertine van Oord, Gabrielle B. A. van Tilburg, Ellen C. Zwarthoff

**Affiliations:** Department of Pathology, Josephine Nefkens Institute, Erasmus MC, Rotterdam, The Netherlands; Université Paris-Diderot, France

## Abstract

Retinoic acid receptor (RAR) signaling is important for regulating transcriptional activity of genes involved in growth, differentiation, metabolism and reproduction. Defects in RAR signaling have been implicated in cancer. TEL, a member of the ETS family of transcription factors, is a DNA-binding transcriptional repressor. Here, we identify TEL as a transcriptional repressor of RAR signaling by its direct binding to both RAR and its dimerisation partner, the retinoid x receptor (RXR) in a ligand-independent fashion. TEL is found in two isoforms, created by the use of an alternative startcodon at amino acid 43. Although both isoforms bind to RAR and RXR in vitro and in vivo, the shorter form of TEL represses RAR signaling much more efficiently. Binding studies revealed that TEL binds closely to the DNA binding domain of RAR and that both Helix Loop Helix (HLH) and DNA binding domains of TEL are mandatory for interaction. We have shown that repression by TEL does not involve recruitment of histone deacetylases and suggest that polycomb group proteins participate in the process.

## Introduction

Nuclear receptors (NR) belong to the large family of well studied transcription factors that are important for growth, differentiation, metabolism and reproduction in higher organisms. Small molecules, such as steroids, thyroid hormones and retinoids serve as ligands and bind to the ligand binding domains (LBDs). NRs bind DNA of target promotors as hetero or homo dimers using their highly homologous DNA binding domain (DBD) [1]. One of these NR, the retinoic acid receptor (RAR) has several isoforms, forms a heterodimer with retinoic-x-receptor (RXR), and binds the ligand *all-trans* retinoic acid (ATRA). RAR-signaling induces differentiation and apoptosis in a wide variety of cells. Furthermore, retinoic acid has tumor-suppressive activity and defects in RAR signaling are implicated in cancers [2], [3].

The regulation of gene expression by NR involves the release and binding of co-repressor and co-activator complexes. Nuclear receptor co-repressor (N-CoR) and silencing mediator of retinoid and thyroid hormone receptors (SMRT) are co-repressors that associate with RAR and recruit complexes with histone deacetylase (HDAC) activity [4], [5]. Activation of RAR target genes involves the binding of co-activators of the p160 family (SRC1, SRC2 also known as Tif2, and SRC3 also known as RAC3) of which most of these have intrinsic histone acetylase (HAT) activity. In addition, HAT activity-containing p300/CBP proteins are recruited by these co-activator complexes [6], [7]. Recent studies have shown that both active and repressed RAR-regulated genes continuously exchange co-activator and co-repressor complexes [8], [9]. This dynamic and cyclic process cause a continuous recruitment of both HAT and HDAC activity to the promoters and the balance between these complexes finally results in either activation or repression of gene expression. In addition, various other processes including ubiquitination, sumoylation, methylation and phosphorylation have been implicated in regulation of NR activity [9], [10], [11], [12].

Here we describe a novel mode of repression of RAR signaling. The repression involves the binding of the transcriptional repressor TEL to RARα and RXRα. TEL (ETV6) is a member of the ETS family of transcription factors. TEL is expressed throughout the body including the hematopoietic system and is crucial for hematopoietic stem cell maintenance, as has been shown in a *Tel* knockout mice model [13]. These mice have been shown to die due deficient yolk sac angiogenesis. The classical mode of repression by TEL has been studied extensively and involves the binding of TEL to DNA-responsive elements within promoters with its DBD domain. The helix-loop-helix (HLH) domain, also called Pointed (PNT) or SAM domain is important for polymerization of TEL [14], [15]. Repression involves either the recruitment of co-repressor complexes and HDACs [16], [17], or the recruitment of L(3)MBT-containing polycomb group-complexes [18] that facilitate long-term repression by chromatin remodeling other than deacetylase activity. Here we show that the interaction between TEL, RARα and RXR involves both DBD and HLH domains of TEL and the DBD domain of RARα. Furthermore, we show that both isoforms of TEL, generated by the use of an alternative start codon, influence RAR signaling, that this repression is HDAC-independent, and that the shorter isoform is a much more efficient repressor compared to the larger isoform.

## Methods

### Constructs

All expression constructs are derived from the CMV promoter containing expression vector pcDNA3. TEL, MN1 and MN1-TEL constructs have been described previously [19], [20]. The DBD mutant of TEL was described elsewhere [21] and was recloned in a pcDNA3 vector. The point mutation in the HLH domain in TEL was introduced using site-directed mutagenesis (Quikchange mutagenesis kit, Stratagene). The primerset used was 5′-cctcattcaggtgatgcggcctatgaac tccttcagc-3′ in combination with a primer that consists of the opposite strand. The DBD mutation was combined with the HLH mutant by ligating the *Bst*EII/*Sca*I fragment from a pcDNA3 TEL-DBD mutant construct into the corresponding sites of the digested HLH mutant constructs. The HA-tagged version of TEL was created by ligating a double stranded oligo containing the DNA sequence for a spacer encoding amino acids GAGAGA followed by the HA sequence and a stop codon, in frame with the last amino acid of TEL. TEL constructs with a forced start at methionine 1 or 43 were created using the Quikchange mutagenesis kit by changing the methionine into a cysteine. Deletion constructs of TEL were generated by PCR using primers containing restriction sites enabling cloning and fusion of the specified regions. The RARE-luc reporter vector contains 3 retinoic acid responsive (DR5) elements in a basic TK luc reporter vector and was a kind gift of Dr. J.Jansen (Dept. of Hematology, Erasmus MC, Rotterdam, the Netherlands). The MSV-luc reporter construct was previously described [20] and the RARE3-MSV reporter was created contains three RARE elements cloned in front of 131 bp of the original MSV promoter. The putative ETS element within the remaining sequence, TTCC, was mutated into TTTT using the Quik Change Mutagenesis Kit. Plasmid pSG5-TIF2 was obtained from Dr. G. Jenster (Urology, Erasmus MC, Rotterdam, the Netherlands) and GST-RAR, GST-RAR-LBD, GST-RXR and GST-RXR-LBD constructs were a kind gift of Dr. H. Gronemeyer (IGBMC, Strasbourg, France). Additional deletion constructs of GST-RAR were generated by subcloning using restriction sites available in the RARα sequence. A bioV5-tagged version of deletion construct N3 of RAR was created by linking the bioV5 sequence [22] at the N-terminus of RAR-N3. pSG513-BirA plasmid for expression of the BioLigase was a kind gift of T.B. van Dijk (Dept of Cell Biology, Erasmus MC, Rotterdam, the Netherlands) [23]. All constructs were checked by DNA sequencing (BigDye Terminator kit, Stratagene) and, in case of expression vectors, also by *in vitro* transcription/translation to confirm that translation products of the correct length were obtained (TNT Quick Coupled Transcription/Translation system, Promega, Madison, WI, USA).

### Transfection

The Hep3B cell line (human hepatocarcinoma) [24] was cultured in α-MEM, supplemented with 5% (v/v) fetal bovine serum and antibiotics (penicillin and streptomycin). Hep3B cells were seeded at a density of 0.6·10^5^ cells per well in 24-well culture plates. 24 hours after seeding the cells the medium was replaced with fresh medium, containing *all-trans* retinoic acid (ATRA, Sigma Aldrich, St. Louis, MO, USA), when indicated, at a concentration of 1 µM. After 1–2 hours the cells were transfected with the indicated amounts of DNA, using FuGENE 6 Transfection Reagent (Roche Applied Science, Basel, Switserland), according to the manufacturer's instructions. In each experiment, the total amount of transfected DNA as well as the molar amount of expression plasmid was kept constant, by addition of the required amount of pUC6 and empty pcDNA3 plasmid. When applicable, three hours after transfection Trichostatin A (TSA; Sigma Aldrich) was added to the specified concentration from ethanol stocks. 24 or 48 hours after transfection luciferase activities were measured in lysates on a Fluoroscan Ascent FL luminometer (Labsystems, Helsinki, Finland).

### GST pull down

GST fusion constructs were expressed in *E. coli* and the fusion proteins were loaded onto glutathion sepharose beads (GE Healthcare, Uppsala, Sweden) according to the manufacturer's recommendations. The amount of GST protein (µg) used for loading was similar for the different fusion proteins in each experiment. When necessary, because of varying levels of GST fusion protein expression, untransformed *E. coli* lysate was added during loading of the beads, to keep also the total amount of lysate (µg protein) at similar levels. Binding of proteins to the immobilized GST fusion proteins was carried out as described by Van Wely et al [25] with the exception that instead of using columns, the beads were incubated and washed in microfuge tubes and collected by centrifugation at maximal speed. Beads containing non-fused GST were used as controls. *In vitro* transcription/translation products labeled with ^35^S-methionine were used for the binding experiments. The bound translation products were eluted from the beads by boiling in SDS-PAGE sample buffer, separated by SDS-PAGE, and visualized by autoradiography.

### Immunoprecipitation

2·10^6^ Hep3B cells were transfected with pcDNA3, pcDNA3 TEL-HA M1C or pcDNA3 TEL-HA M43C in combination with pcDNA3 BioV5-RARN3 and BirA plasmids using Fugene 6 transfection reagent (Roche) according to manufacturer's protocols. 48 hours after transfection, cells were harvested and subsequently lysed using a buffer containing 20 mM Tris pH 8.0, 137 mM NaCl, 10% glycerol, 1% NP-40, 2 mM EDTA (pH 8.0) supplemented with Complete Mini protease inhibitor cocktail 9 (Roche). BioV5 RAR-N3 proteins were precipitated using streptavidin-coated Dynabeads (Invitrogen Dynal AS, Oslo, Norway). Precipitated proteins were subjected to SDS PAGE and Western blotting according to standard protocols. Antibodies: a-TEL, clone 3B10 (Abnova, Taipei, Taiwan); a-HA, biotin-tagged, clone 3F10 (Roche); streptadivin-HRP, (BioGenex Laboratories Inc., San Ramon, CA, USA)

## Results

### TEL binds directly to RARα and RXRα

**Figure 1:**
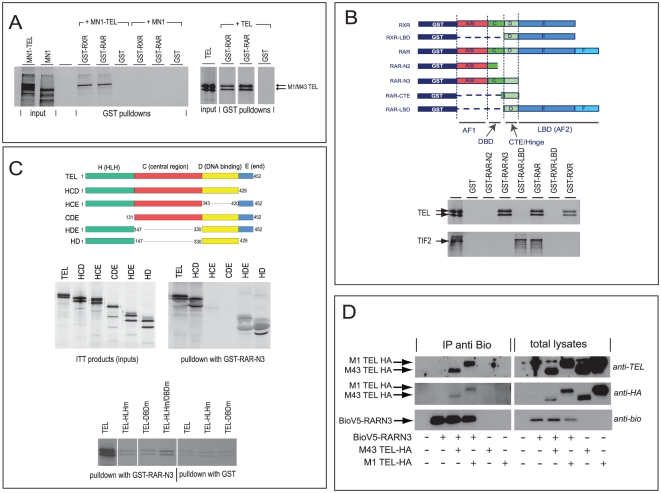
TEL binds RAR and RXR *in vitro* and *in vivo*. (A) TEL and MN1-TEL bind RAR and RXR whereas MN1 does not. *In vitro* generated, 35S-labeled proteins, as shown in the input lanes, were used for GST pulldowns with GST-RAR, GST-RXR and GST (as control). (B) TEL binds the DBD domain of RARα. Upper panel: Schematic overview of the RARα and RXRα constructs. The domains within RARα and RXRα are indicated with letters commenly used to indicate the domains within the nuclear receptor family. At the bottom the different regions are indicated with respect to their function. AF1, Activator function 1; DBD, DNA binding domain; CTE, C-terminal extension; LBD, ligand-binding domain; AF2, activator function 2. Lower panel: GST pulldowns with *in vitro* transcribed/translated TEL and TIF2. Binding of TEL to RAR and RXR differs from the binding of TIF2 to RARα and RXRα. Binding of TIF2 was detected for LB-containing RARα constructs whereas TEL bound to the DBD region of RARα. No binding of TEL to the AF1 and first part of DBD was detected (GST-RAR-N2). (C) HLH and DBD domains of TEL are crucial for binding to RARα. Upper panel: Schematic overview of deletion constructs of TEL. Numbers indicate the amino acid positions. Middle left: ITT products were used for GST pulldown assays with GST-RAR-N3. Middle right: Only deletion constructs that contain both HLH and DBD domains were detected in the in vitro binding assay. Lower panel: WT TEL showed binding to GST-RARα, whereas HLH, DBD or double mutant TEL proteins failed to bind GST-RARα. (D) bio-precipitation of bioV5-tagged RAR-N3 proteins. Only in the presence of bioV5-tagged RARN3, TEL-HA proteins were detectable in the precipitations, showing the *in vivo* binding between TEL and RAR. Right panel shows input lysates. TEL proteins were detectable with both a-TEL and a-HA antibodies.

The transcriptional cofactor MN1 stimulates and inhibits RAR/RXR-mediated transcription [26]. In order to investigate if MN1 bound to RARα and/or RXRα we performed GST pulldown experiment using GST-fused RARα (GST-RARα) and GST-RXRα together with *in vitro* produced and 35S-labeled proteins. MN1 was unable to bind to GST-RARα and RXRα (Figure 1A). Other cofactors are thus involved in this transcription regulation. In this experiment we also tested the MN1-TEL protein. This leukemogenic protein is formed by an AML-causing translocation (12;22) between *MN1* and *TEL* genes. To our surprise, MN1-TEL bound very strongly to RARα and RXRα. In order to locate the domain that binds RARα and RXR we investigated whether binding was a property of the TEL moiety in the MN1-TEL protein. This indeed appeared to be the case as is shown in figure 1A, right panel. The presence of an alternative start methionine at aa 43 of the coding region of the *TEL* gene gives rise to two isoforms of TEL [27], [28]. Both isoforms of TEL bound GST-RARα and GST-RXRα equally efficiently in this GST pulldown assay.

### TEL binds to the DBD domain of RAR whereas the coactivator Tif2 binds to the ligand binding domain

Tif2, also known as SRC2, is a coactivator of RAR that binds the ligand binding domain (LBD) in a ligand dependent fashion [7]. The LxxLL motif of Tif2 provides the binding interface. TEL also harbors a LxxLL motif (aa 113–117, LYELL) within the HLH domain and this prompted us to investigate the possibility that Tif2 and TEL are displaying similar binding characteristics. We obtained and generated deletion constructs of RARα and RXRα that harbor the different regions of the receptor (Figure 1B). Pulldowns using these constructs showed that Tif2 could bind to RARα but not to RXRα. TEL interacted with both RARα and RXRα constructs. The RARα and RXRα deletion constructs showed that TEL interacted with RARα constructs that contain the AF1 and DBD regions of RARα, whereas Tif2 interacted with the LBD region (Figure 1B). The binding of TEL was independent of the ligand ATRA, whereas the binding of Tif2 was lost if ATRA was not available (data not shown). The smallest RARα construct tested only contains the CTE region of the DBD. This construct was efficiently expressed but TEL failed to bind this region (data not shown). From these experiments we conclude that the binding of TEL to RARα and RXRα is within or close to the DBD domain of RARα and that it differs from the binding of Tif2 to RARα, which is ligand-dependent.

### Both the HLH domain and the DBD of TEL are required for binding

The TEL protein is member of the ETS family of transcription factors that have several domains in common. We have generated deletion constructs to investigate which domains of the protein are crucial for binding to RARα. A schematic overview is shown in Figure 1C. All constructs were efficiently produced in the *in vitro* transcription/translation system (middle left panel of Figure 1C) and were used in pulldown experiments (middle right panel of Figure 1C). All constructs that contain both the HLH and DBD domains of TEL were binding to RARα whereas other constructs failed to bind. Both HLH and DBD domains of TEL are thus crucial for binding to RARα. It has also been described that the HLH domain of TEL causes aggregation of TEL *in vitro* and this could result in entrapping of other proteins. Such a a-specific entrapping of proteins does not explain the interactions shown in Figure 1 since (1) we do not see interaction with GST, and (2) we also do not see any interaction with two TEL mutants in which the polymerization domain is still intact, i.e. the TEL-DBDmutant and the deletion mutant HCE.To investigate whether also small point mutations within the DBD and HLH domains of TEL can abrogate the interaction between TEL and RARα we generated two mutants of TEL. The mutations within the HLH domain of TEL (V112A/L113A) are located in the binding surface of the HLH structure important for polymerization. This mutant is impaired in polymerization (data not shown). The DBD mutant of TEL (R396L, R399L) was described previously [21], [29] and fails to bind ETS responsive elements. In our *in vitro* binding assay with GST-RARα both TEL mutants were tested for interaction with RARα. Figure 1C, lower panel shows that these TEL mutants failed to bind RARα. From this we conclude that both HLH and DBD domains are crucial for binding to RARα and that mutations that impair the function of these domains also disrupt binding of TEL to RARα.

### In vivo binding of TEL and RAR

To examine the interaction between TEL and RAR *in vivo*, we transfected Hep3B cells with an expression plasmid of N-terminal part of RARα (RAR-N3) tagged with a V5 and bio tag at the N-terminus and a M43-TEL-HA or M1-TEL-HA construct. RAR proteins were precipitated on the bio tag using streptavidin beads and precipitates were subjected to immunoblotting for TEL proteins. TEL-HA proteins, detected with either an antibody against TEL or the HA-tag, co-precipitated with RAR-N3 protein. No signal for TEL proteins was observed in negative control precipitations (Figure 1D).

### M43-TEL represses transcription directed by the nuclear receptor dimer RAR/RXR

**Figure 2:**
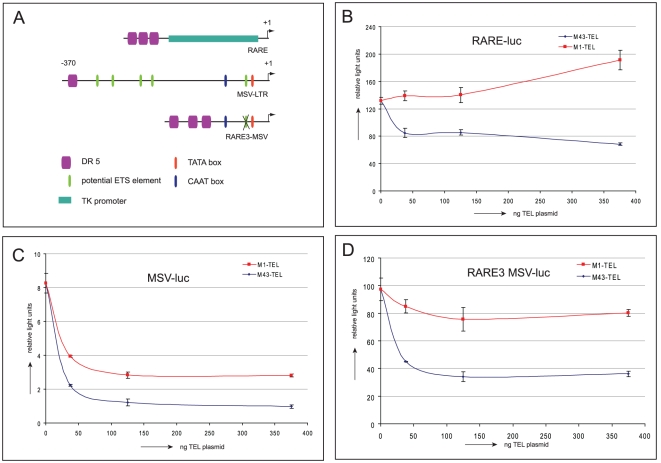
TEL represses transcription that is mediated by the heterodimer RAR/RXR. (A) Schematic overview of different luciferase reporter constructs RARE-luc, MSV-luc and RARE3MSV-luc. (B) The M43-TEL isoform inhibits the RARE luc reporter whereas the M1-TEL isoform does not. (C) The viral promoter MSV is repressed by both isoforms of TEL. M43-TEL is more efficient. (D) Both TEL isoforms repress the RARE3 MSV-luc construct. M43-TEL is more efficient. All transfections were performed in the presence of *all-trans* retinoic acid (ATRA).

The binding of TEL to RARα and RXRα prompted us to investigate if TEL can also repress transcription that is regulated by the nuclear receptor heterodimer RAR/RXR. In Hep3B cells, the retinoic acid-responsive element (RARE) from the RARβ promoter cloned in front of a TK promoter (Figure 2A) was strongly stimulated by ATRA (data not shown), which activates endogenously expressed RAR and RXR. Although both isoforms of TEL are binding to RARα and RXRα, only the shorter isoform (M43-TEL) exhibited repression activity on this promoter (Figure 2B) in this assay. The moloney sarcoma virus long terminal repeat (MSV-LTR) is a viral promoter that is regulated by numerous transcription factors [30], [31]. We have shown previously that the MSV promoter harbors a genuine RAR/RXR-responsive element (RARE), also known as a direct repeat 5 (DR5) element [25]. The remainder of the sequence has at least five potential ETS-responsive elements that possibly represent target sequences for TEL. Both isoforms of TEL strongly repressed the activity of the MSV-LTR although the shorter isoform (M43-TEL) was more efficient (Figure 2C). The extent of the repression was much greater compared to the repression observed on the RARE luc reporter. We hypothesized that on this viral promoter, the repressive effects on ETS elements are combined with repression by TEL via the RAR/RXR dimer. In order to investigate repression on the RARE element in the context of the MSV-LTR, we juxtaposed three copies of the RARE element from the MSV promoter adjacent to the 3′ region of the MSV-LTR, which harbors crucial elements like the TATA box and CAAT box, thereby deleting all but one ETS element. This remaining putative ETS element, which is located between the TATA and CAAT boxes, was removed by mutating two C's into 2 T's (Figure 2A). The resulting promoter, called RARE3-MSV, was repressed by TEL and the extent of the repression was lower compared to the original MSV-LTR constructs (Figure 2D) but somewhat higher when compared to the RARE luc constructs. The larger isoform of TEL (M1-TEL) still had some residual repression on this construct.

### Besides HLH and DBD domains, other regions of TEL are important for repression of RAR/RXR-mediated transcription

**Figure 3:**
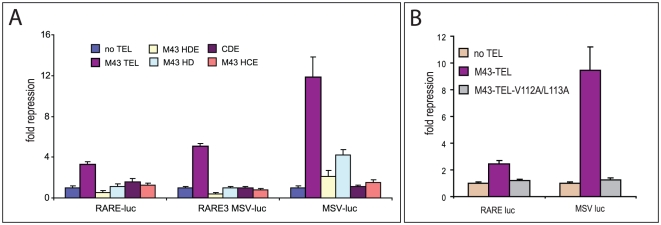
Deletion mutants of TEL have no repressive activity. (A) Three different reporters were tested with TEL deletion mutants. The repression of TEL on the RAR-RXR dimer (i.e. RARE luc and RARE3-MSV luc) was only detected using full length M43 TEL. Although deletion mutant HD was able to bind RAR in vitro (figure 1), it was unsuccessful in repression. The MSV-luc reporter contains not only an RAR/RXR responsive element but also many possible ETS binding sites (figure 2E). The HD deletion construct of TEL repressed the MSV-luc reporter efficiently. (B) Both modes of repression by TEL (i.e. on RAR/RXR and on ETS sites) are disabled in the HLH mutant of TEL (TEL-V112A/L113A).

We next tested the ability of the TEL deletion mutants and point mutants to repress the activity of the MSV LTR and the RARE reporter plasmids in Hep3B cells (Figure 3A). The classical mode of repression by TEL is conducted on the MSV reporter and involves the binding to ETS elements. Although the HD deletion mutant lacks the central region of the TEL protein, that is crucial for the recruitment of corepressors N-CoR and SMRT [16], [17], [32], it still was able to repress transcription of the MSV-LTR. Either recruitment of mSIN3A via the HLH domain is sufficient for repression or dimerization with other intact TEL molecules that are endogenously expressed in Hep3B cells provides the interface for recruitment of other co-repressors. In contrast, the repression of RAR-RXR-responsive elements fully depends on regions other than the HLH and DBD domains of TEL. Whereas M43-TEL inhibited the activity of the RARE luc or RARE3-MSV reporter, none of the tested deletion mutants did. The results of the DBD mutants were inconsistent. DBD deletion mutants were unable to repress the reporters whereas TEL that contains point mutations within the DBD (TEL-DBDm) was capable of low-level repression (data not shown). TEL proteins that either contain a point mutation in or a deletion of the DBD domain localized exclusively to the cytoplasm and therefore their effect on RAR/RXR-induced expression could not be tested. Next we investigated if the HLH mutant construct, which is unable to polymerize with itself, but showed some residual polymerization with wildtype TEL (data not shown), was able to carry out either mode of repression. Figure 3B shows that this mutant was unable to repress any of the two reporters. From this we conclude that dimerization of TEL proteins is important for repression on ETS elements that are present in the MSV_LTR.

### Repression of RAR/RXR-mediated transcription is HDAC independent

**Figure 4:**
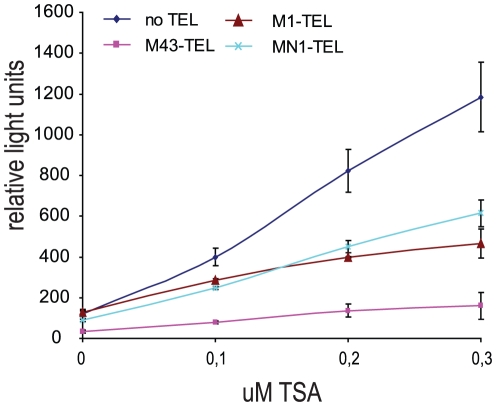
Repression of TEL on RAR/RXR-mediated transcription does not involve histone deacetylase activity. Increasing amounts of TSA were tested on RARE luc reporter in the presence of ATRA, and with and without M1-TEL, M43-TEL or MN1-TEL (120 ng). RAR/RXR-mediated transcription was stimulated by TSA to high levels, whereas M43-TEL prevents this. Intermediate effects were observed with MN1-TEL and M1-TEL.

The recruitment of co-repressor complexes that subsequently interact with histone deacetylases (HDACs) is one of the ways in which TEL represses transcription when it binds to TEL responsive elements. Whether such mechanisms are also involved in the repression of RAR/RXR-mediated transcription by TEL was tested using trichostatin (TSA). TSA is a strong inhibitor of deacetylases and is expected to abrogate repression by TEL if repression is carried out by deacetylation of histones. Increasing amounts of TSA were tested on the RARE luc reporter. In the absence of TEL, TSA strongly increased the activity of the promoter, suggesting that HDAC-dependent down regulation of expression is inhibited and the promoter remains active. In the presence of M43-TEL, however, RAR induced expression is inhibited and TSA hardly increased the activity, suggesting that the repression of RAR/RXR-mediated transcription by TEL depends on other repressive mechanisms than histone deacetylation (Figure 4). Although both M1-TEL and MN1-TEL proteins are able to bind RAR (Figure 1), no direct repression was observed on the reporters (Figure 4, 0 µM TSA and Figure 2B). In the presence of TSA both proteins however showed intermediate effects: the activity of the promoter is far less stimulated by TSA. This suggests that both M1-TEL and MN1-TEL have repressive capacity although at a lower level compared to M43-TEL.

## Discussion

RAR-RXR signaling and TEL expression are required for proper embryonic haematological development. In adult life these proteins are expressed throughout the body and regulate various biological processes including cellular proliferation, differentiation, hematopoiesis, angiogenesis and transformation [1], [33], [34]. RAR-RXR regulated gene expression involves the dynamic and continuous binding and release of co-repressors and co-activators. Activation of RAR-RXR is the result of recruitment of co-activators to the receptors upon binding of the ligand. The co-activators recruit p300 a histone acetyl transferase or HAT. Acetylation of histones then facilitates gene expression. Subsequent down regulation is by exchange of the co-activators for co-repressors and these in turn recruit histone deacetylating proteins (HDACs) [8]. In the presence of ligand only a small fraction of the RAR-RXR target promoters is active as can be deduced from the finding that inhibition of HDAC activity by TSA results in an at least 8-fold increase in expression from the reporter as indicated in Figure 4. Thus TSA apparently fixes the reporter gene in the active state and consequently the pool of active reporter genes is much larger than in the presence of ATRA alone. Here, we provide evidence that TEL (ETV6) is a co-repressor for RAR. The interplay between TEL and RAR has not been described before, as far as we are aware. The TEL-M43 isoform actively represses transcription of the reporter by RAR-RXR. In the presence of TSA the activity of the reporter is only marginally increased. The other TEL forms, TEL-M1 and the fusion protein MN1TEL also bind RAR-RXR and they also reduce the effect of TSA. This suggests that inhibition of RAR-RXR activity by TEL is HDAC independent and in fact replaces HDAC activity. It is tempting to speculate that this property of TEL is due to the recruitment of polycomb repressor complexes through TEL's binding partner L(3)MBT. Alternatively it is possible that TEL prevents RAR binding to DNA, similar to Oct-1 and Myb. Further studies should establish which mechanism is used by TEL.

The human tumor antigen PRAME (PRreferentially expressed Antigen in MElanoma) and HACE1 (HECT domain and Ankyrin repeat containing E3 ubiquitin-protein ligase) were both recently identified as a co-repressors of RAR signaling [35], [36]. These co-repressors also function via HDAC-independent mechanisms and PRAME was shown to interact with polycomb group protein EZH2. Despite the interesting similarity between HACE1, PRAME and TEL as co-repressors with respect to HDAC-independent repression, many differences are also apparent: (1) PRAME binds RAR only in the presence of its ligand ATRA, whereas TEL and HACE1 bind both in the presence or in the absence of ATRA, (2) PRAME binds the LBD of RAR and HACE1 binds the N-terminal activation function 1 (AF-1) domain whereas TEL binds the DBD of RARα, and (3) PRAME and HACE1 do not bind RXR, whereas TEL also binds RXRα.

We have shown that two well-studied domains of TEL, the HLH and DBD domains, are mandatory for the interaction. Even small point mutations in either of the domains abrogates the binding. The binding of TEL to RARα involves the DBD of RARα and over the last years a growing number of co-regulators have been identified that bind this highly homologous region of the NRs. The origin of these regulatory proteins is diverse. RIF1 and HET/SAF-B are nuclear matrix proteins and both were shown to interact with the DBD region of the estrogen receptor (ER). In addition, RIF1 was shown to bind the DBD of RAR and the glucocorticoid receptor (GR) [37], [38]. Binding of RIF1 translocates RAR to the nuclear matrix, thereby limiting the amount of RAR available for DNA binding, resulting in repression of RAR-induced transcription. Oct-1 and Myb are transcription factors that bind to the DBDs of RXR, RAR, the vitamin D (VD) and thyroid hormone receptor (TR) [39], [40]. Both proteins prevent the NR dimers to bind DNA, thereby reducing the fraction of transcriptionally active NRs. In addition, Schick et al [41], have described TEL as a repressor of Stat3 (Signal Transducer and Activator of Transcription 3) signaling. TEL binds Stat3 and DNA binding is not required. Stat3 repression by TEL is HDAC-dependent, and thus different from inhibition of RAR-RXR-mediated transcription by TEL.

In summary, we have identified TEL (ETV6) as a co-repressor of RAR-RXR-mediated transcription. The binding of TEL to RXRα suggests that TEL may broadly interact with NR signaling, since RXR also dimerizes with the TR, VDR, peroxisome proliferator-activated receptor (PPAR) and a variety of other orphan receptors [42]. If and how TEL represses other NR signaling pathways are questions to be answered in the future. We hypothesize that TEL bypasses deactivation of a promoter by HDACs perhaps by recruiting polycomb complexes through one of the L(3)MBT proteins. Thus TEL has been added to the growing list of NR co-regulators and since both TEL and RAR signaling are implicated in a broad spectrum of biological processes, both in health and disease, this interaction might be important to understand some of the biological effects of TEL and RAR.
